# COVID-19 pandemic: different roles for scientific publications and funding face to epidemiological data—an European, country-based perspective

**DOI:** 10.1186/s12948-021-00154-9

**Published:** 2021-09-13

**Authors:** Alessandro Tonacci, Sara Genovese, Giovanni Pioggia, Sebastiano Gangemi

**Affiliations:** 1 Institute of Clinical Physiology, National Research Council of Italy (IFC-CNR), Via Moruzzi 1, 56124 Pisa, Italy; 2 Institute for Biomedical Research and Innovation, National Research Council of Italy (IRIB-CNR), Messina Unit, Via Vincenzo Leanza, Mortelle, 98164 Messina, Istituto Marino Italy; 3 School and Operative Unit of Allergy and Clinical Immunology, Department of Clinical and Experimental Medicine, University Hospital “G. Martino”, Via Consolare Valeria SNC, 98125 Messina, Italy

**Keywords:** Coronavirus, COVID-19, Data analysis, Epidemiology, Pandemic

## Abstract

COVID-19 has represented an unprecedented challenge to be faced also concerning the spread of information, with scientific literature being often the sole source of trustworthy knowledge for the global community. However, a massive waste in research was noticed during pandemic, preventing the scientists to produce totally novel and original results, and the citizenship to have the complete support they needed from science. The present work investigated the relationship between planned funding, research grants, scientific publications and epidemiology in the 27 EU countries, retrieving a significant correlation between scientific publications and COVID-19 cases and deaths, as well as with economic data. Interestingly, planned coronavirus-devoted funds were correlated with lower GDP per capita and higher mortality, leading to the hypothesis for a lack of translation into real funds allowed to the respective country, or for a significant research waste, not transformed into a tangible product or effect. Such results could suggest the need for a different approach in the future concerning the redistribution of research funds in case of COVID-19 relapse or future pandemic events.

## Background

The COVID-19 pandemic has represented an unprecedented threat to the global safety and security, affecting any country in the world, with dramatic consequences in terms of life losses, burden for healthcare systems and economic stability of the planet. In this regard, science has often been the sole reliable response to the pandemic, both in terms of literacy for the community and citizenship at large, challenging the mounting threats of the “fake news” spread [1, 2], and in terms of developing new strategies for effectively switching off the pandemic.

Vaccines are nowadays the most largely employed strategy for that purpose and, apparently, the most efficaceous mean of combating the COVID-19 related infection [3], with their development in such a short timeframe having required a strong, synergistic effort by pharma companies, governmental bodies and research funders. Funding scientific research on COVID-19 has been one of the ways countries and transnational funding bodies have had to attempt blocking the pandemic spread; however, data concerning the amount of funds received by each country and their relationship with epidemiological, country-based data on the COVID-19 has not been investigated, to date. At the same time, it has not been investigated how much, foreseen funds granted on a country basis, has been translated into a scientifically relevant impact and, ultimately, to a detectable effect on the population at large, later on.

Therefore, the present work has tried to merge together epidemiological data about COVID-19 with those concerning scientific publications, funds expected to be granted and real grant funding in European countries, in order to understand whether a correlation occurs among such different data.

## Investigation

To carry out the investigation, we used open-access, easy-to-retrieve data referring to the pandemic impact around European countries, including the number of cases, cases per million inhabitants, deaths, deaths per million inhabitants and mortality, calculated as the ratio between deaths and cases. Also, we used socio-economic data according to the open access Wikipedia databases. We compared them with the COVID-19-related scientific production and grant funding. More in depth, data concerning scientific production was obtained by searching the PubMed database for papers published until April 18th, 2021, according to the following string: (“covid-19” or “coronavirus" or "covid" or "sars-cov-2" or "sars-cov2") and ("COUNTRY" [LocationID]). Planned funds were retrieved from Statista (https://www.statista.com/statistics/1104167/eu-27-eu-funding-to-fight-coronavirus-by-country/), whereas data concerning grant amounts devoted to research activities per country were retrieved from the COVID-19 Research Project Tracker by UKCDR & GloPID-R) https://www.ukcdr.org.uk/covid-circle/covid-19-research-project-tracker/).

Both data about the scientific production and planned funds/research grants were considered as both the overall number of papers (or amount of money) published dealing with COVID-19 and considering a given country, and its ratio per million inhabitants of the selected country.

For the present study, statistical correlations were performed by SPSS v.23 (IBM Corporation, Armonk, NY, USA) using a two-tailed Spearman’s Test. Bonferroni post-hoc analysis was then applied to retrieve significant results, considered to be those with p < 0.05. This choice was performed taking into account the low sample size of the dataset employed and the need for minimizing the false positive results in terms of correlations retrieved.

The 27 EU countries were included in this study.

After the Bonferroni correction, the total number of publications were directly correlated with: i) total number of cases (r = 0.739, p < 0.001), ii) total number of deaths (r = 0.659, p < 0.001), and inversely with planned funds pro capite (r = − 0.565, p = 0.002). When considering the number of publications per inhabitants, they were directly related to the Gsross Domestic Product (GDP) per capita (r = 0.711, p < 0.001), and inversely to the planned funds pro capite (r = − 0.681, p < 0.001).

When it comes to the planned funds, they were positively correlated with: i) total number of deaths (r = 0.589, p = 0.001), ii) deaths per million people (r = 0.530, p = 0.004), iii) mortality (r = 0.694, p < 0.001), and negatively related to: i) publications pro capite (r = -0.600, p = 0.001), and ii) GDP per capita (r = − 0.706, p < 0.001). Finally, the planned funds pro capite were negatively correlated to the GDP per capita (r = − 0.818, p < 0.001). All the correlations are displayed in Table 1.

**Table 1:**
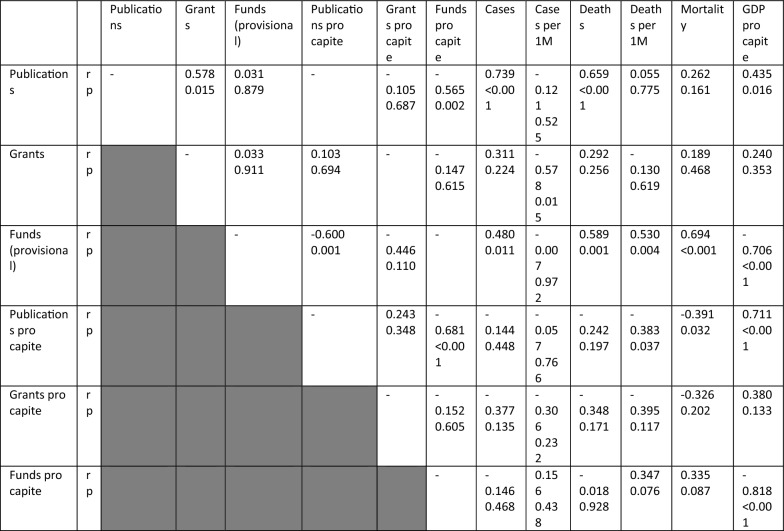
Correlations between epidemiological and scientific production-related parameters

The COVID-19 awareness is pivotal to effectively fight the direct and indirect consequences of the misleading information for scientists and the general public. However, the number of scientific products related to the COVID-19 was seen to be higher in those European countries where the pandemic brought the greater burden, or in some cases in regions where a better economic status was seen. This is somewhat similar to the results displayed worldwide concerning the relationship between COVID-19 spread and socio-economic status of a country [4]. In that case, the countries more at risk for higher occurrence of COVID-19 cases were those with higher socio-economic status where, apparently, people were spending more time within indoor, overcrowded environments, or where international mobility, including more frequent flights per inhabitant, was more frequent. Those countries, even in a more geographically limited and socio-economically uniform scenario like the European continent, are also more likely to have resources and infrastructure to produce a wider number of scientific articles, therefore justifying the results obtained in the present research.

Despite the shorter time needed during pandemic to obtain scientifically relevant and detectable results with respect to the past, the amount of research grant funds obtained by the European countries analyzed in the present work did not appear to be correlated to a detectable effect at a country level based on epidemiological data. This is possibly due to several reasons, among which the fact that nationally awarded grant do not necessarily reflect on merely single country-based data, but could also produce output values which are transferrable beyond the national borders. Another reason for this lack of statistical correlation involves the timeframe considered, since some funded research could produce significant outputs in terms of reduction of morbility or mortality even in longer time windows, for which actual data are not indicative, yet.

On the other hand, analyzing the amount of planned funds and its relationship with the epidemiological data, it comes to the eye that the countries having foreseen a larger amount of money to be awarded were those with the worst economic situation, and had the lower impact in terms of scientific production. Furthermore, the planned funds resulted to be of scarce impact in terms of quenching the pandemic spread.

Such resulting evidence could be due to several different reasons. It could be hypothesized that at least part of the planned funds were not delivered to the countries as initially foreseen, therefore not producing any positive output either in epidemiological and scientific terms, not allowing poorer countries to perform a significant scaling-up of their infrastructure and activities to properly tackle the pandemic. On the other hand, it could be supposed that a significant amount of money could have been driven to a poor quality of research product, feeding the so-called “waste in research”, phenomenon now stronger than in the past due to the pandemic-inspired rush to research [5]. Such eventuality should be carefully taken into account in this particular period to properly overcome the COVID-19 pandemic and future events.

Despite the massive efforts carried out by the scientific community to make research reliable, appropriate and useful to challenge the COVID-19 pandemic, when it comes to the European continent, a greater amount of country-based scientific products and grants was not enough to properly support a country tackling the pandemic spread. Possibly positive effects brought by funding, production and access to science in a given country are probably counterbalanced either by the lack of sufficient quality of the research infrastructure in poorer countries, or by the daily habits of wealthier inhabitants for a more intense social life, especially within indoor locations, in richer nations. The rush to research is another phenomenon to be seriously considered, as it threatens the reliability of scientific data production, leading to poor quality of related research. Future policies should be undertaken in this field, ensuring a higher quality of scientific products, with a rigorous peer-review process to avoid spreading biased results feeding considerable waste in research related to the COVID-19 and possible future pandemic occurrences. In addition, when it comes to fund allocation, they should not be awarded just based on the GDP or similar economic parameters, paying more attention to less developed countries. Conversely, longer-term perspectives should be considered, allowing poorer countries to build up infrastructures and networks in a timely manner, and wealthier regions to take full advantage of their higher readiness and infrastructure level to effectively tackle future pandemics. As such, based on the affordability of the different measures to tackle the pandemic, poorer countries, less likely to have the chance to be included in scientifically relevant large consortia, thus not necessarily receving wide amounts of money or not having the possibility to translate such funding into viable research frameworks, should adopt early screening strategies, relying on fast, useful biomarkers for infection detection, in turn being economically affordable. They could span from questionnaires about the quality of life of such people until home-based screening tools for side-effects, that might occur in the asymptomatic population even early during the infection process, therefore blocking the infection chain early [6].

Those lessons can be learned by the COVID-19 experience and should be treasured for future purposes [7].

## Data Availability

The datasets generated and/or analysed during the current study are available in the repositories mentioned within the references [1, 3–5].
